# The risk of common mental disorders in Indigenous Australians experiencing traumatic life events

**DOI:** 10.1192/bjo.2021.1063

**Published:** 2021-12-06

**Authors:** Bushra Farah Nasir, Elizabeth G. Ryan, Emma B. Black, Stephen Kisely, Neeraj S. Gill, Gavin Beccaria, Srinivas Kondalsamy-Chennakesavan, Geoffrey C. Nicholson, Maree Toombs

**Affiliations:** Rural Clinical School, Faculty of Medicine, The University of Queensland, Australia; Centre for Health Services Research, Faculty of Medicine, The University of Queensland, Australia; and QCIF Facility for Advanced Bioinformatics, Institute for Molecular Bioscience, The University of Queensland, Australia; Rural Clinical School, Faculty of Medicine, The University of Queensland, Australia; Princess Alexandra Hospital Southside Clinical Unit, Faculty of Medicine, The University of Queensland, Australia; Rural Clinical School, Faculty of Medicine, The University of Queensland, Australia; and School of Medicine, Griffith University, Australia; School of Psychology and Counselling, The University of Southern Queensland, Australia; Rural Clinical School, Faculty of Medicine, The University of Queensland, Australia; Rural Clinical School, Faculty of Medicine, The University of Queensland, Australia; Rural Clinical School, Faculty of Medicine, The University of Queensland, Australia

**Keywords:** Aboriginal and Torres Strait Islander, Indigenous Australians, trauma, common mental disorders, Structured Clinical Interview for DSM-IV Axis I Disorders

## Abstract

**Background:**

Experiencing traumatic life events is associated with an increased risk of common mental disorders (CMDs), but studies investigating this association within Indigenous populations are limited.

**Aims:**

The aim of this study was to investigate associations between trauma and CMDs after controlling for other exposures.

**Method:**

Trauma exposures and CMD diagnoses were determined in a broadly representative sample of 544 Indigenous Australians, using a diagnostic clinical interview. Associations were determined by multivariate logistic regression.

**Results:**

Trauma exposure independently predicted CMDs. After adjustment for potential confounders, trauma exposure was associated with a 4.01-fold increased risk of a diagnosis of a CMD in the past 12 months. The increased risks were 4.38-, 2.65- and 2.78-fold of having an anxiety disorder, mood disorder or a substance use disorder, respectively. Trauma exposure and comorbid post-traumatic stress disorder was associated with a 4.53-fold increased risk of a diagnosis of a mood disorder, 2.47-fold increased risk of a diagnosis of a substance use disorder, and 3.58-fold increased risk of any diagnosis of a CMD, in the past 12 months. Experiencing both sexual and physical violence was associated with a 4.98-fold increased risk of a diagnosis of an anxiety disorder in the past 12 months.

**Conclusions:**

Indigenous Australians experience significantly increased exposure to potentially harmful trauma compared with non-Indigenous Australians. Preventing and healing trauma exposure is paramount to reduce the high burden of CMDs in this population.

Australia's Indigenous minority (3% of the overall Australian population^1^) experience distinct health disparities compared with their non-Indigenous counterparts. Life expectancy is at least 10 years shorter,^2^ and death before 65 years of age is three to four times more prevalent.^3^ Latest national reports identify that their burden of disease is 2.3-fold higher.^4^ In addition, almost 65% of Indigenous Australians report a long-term health condition, 29% with a self-reported continuing mental health condition.^5^ These ongoing health disparities are associated with determinants such as the intergenerational effects of colonisation and forced assimilation, social injustices, economic deprivation (e.g. poverty, unstable housing) and systemic racism experienced by Indigenous Australians.^6,7^ Moreover, these health gaps may also represent differences between Western and Indigenous concepts of health.^8^ Despite these multiple factors that significantly contribute to their health and well-being, there is still a lack of policies and programmes addressing these gaps for Indigenous Australians.

This study measures the associations between the prevalence of common mental disorders (CMDs) and trauma exposure. We used a structured interview that quantified the presence of psychiatric disorders according to diagnostic criteria from the DSM-IV-TR, the Structured Clinical Interview for DSM-IV Axis I Disorders (SCID-I).^9^ Preliminary research indicates that the interview is culturally safe and relevant as a diagnostic tool suitable for the study population, when administered by psychologists with appropriate cultural training.^10^

## Method

### Study design

This study is part of a larger cross-sectional prevalence study, for which participant recruitment, involvement and other procedures have been previously reported.^11^ Data categories for CMDs^11^ and traumatic life events^12^ have also been previously described. CMD diagnoses were categorised into three major diagnosis classes: mood, anxiety and substance use disorders. Traumas reported were examined and coded into categories consistent with DSM-IV-TR and SCID-I post-traumatic stress disorder (PTSD) criteria. The data and categories were reviewed by two senior clinical psychologists (G.B., E.B.B.) and a senior consultant psychiatrist (S.K.) during extraction, classification and analyses. Events reported as trauma, but falling short of formal definition, were not included in the analyses.

### Ethics approval

The authors assert that all procedures contributing to this work comply with the ethical standards of the relevant national and institutional committees on human experimentation and with the Helsinki Declaration of 1975, as revised in 2008. All procedures involving human patients were approved by The University of Queensland Human Research Ethics Committee (clearance number: 2012001315) and the Boards of Directors of the participating Aboriginal Medical Services. The study was conducted in accordance with the National Health and Medical Research Council of Australia Guidelines (Values and Ethics: Guidelines for Ethical Conduct in Aboriginal and Torres Strait Islander Health Research, 2003). The research protocol was established in consultation and using co-designed engagement processes. Permission from Indigenous Elders was obtained before recruiting from reserves and communities. Written informed consent was obtained from all participants before any data collection or analyses.

### Data analysis

Descriptive analyses of participant demographics (*n* = 544) have been previously reported.^11^ A significant majority were women (62%), and the age distribution of the cohort was similar to that of Indigenous Australians nationwide.^13^ Crude and standardised prevalence estimates of the major classes of CMD for those with and without reported trauma were analysed for current (past 30 days), past 12-month and lifetime prevalence. Results were stratified by gender and age groups. We excluded PTSD as a separate category because those with PTSD have had exposure to trauma by definition.

Univariate and multivariate binary logistic regression analysis were used to determine the risk of a CMD associated with various trauma categories and other exposures. Separate logistic regressions were fitted for the outcomes of anxiety disorder (excluding PTSD), mood disorder, substance use disorder and any CMD (excluding PTSD). Variables with a *P*-value ≤0.1 in the univariate analyses were carried forward into the relevant multivariate logistic regressions. Stepwise regression was performed for the multivariate models for each outcome to determine the final model (based on the Akaike information criterion value). Multicollinearity between the explanatory/risk factor variables was assessed with the variance inflation factor. All models were fitted in R (Microsoft Version 3.6.2 for Windows).

### Patient involvement

Participants of the study were involved in extensive consultations before the study was designed, implemented and evaluated. Procedures for the involvement of participants have been previously described.^11^

## Results

### Prevalence of CMDs

Crude rates for the 30-day, 12-month and lifetime prevalence of CMDs for those with and without reported trauma is described in Table 1. Overall, those experiencing trauma experienced higher prevalence of all CMDs for all periods of assessment. Of those with trauma exposure (*N* = 353), the majority were women (62%); although men with trauma exposure had higher rates for 30-day (52.7%), 12-month (61.8%) and lifetime (84.6%) prevalence of any CMD (Table 1). After standardising against the Australian population,^14^ the incidence of CMDs for those without trauma was low, and so standardised rates using gender stratification was not performed (Appendix 1).

**Table 1:** Crude prevalence of common mental disorders in Indigenous Australians reporting trauma exposure

	Male, *n* (%)		Female, *n* (%)		Total, *n* (%)	
	Trauma (*n* = 133)	No trauma (*n* = 74)	Trauma (*n* = 220)	No trauma (*n* = 117)	Trauma (*n* = 353)	No trauma (*n* = 191)
30-day prevalence						
Mood disorder	27 (20.3)	8 (10.8)	53 (24.1)	10 (8.5)	80 (22.7)	18 (9.4)
Anxiety disorder^a^	31 (25.0)	7 (9.5)	75 (40.1)	15 (12.8)	106 (34.1)	22 (11.5)
Substance use disorder	32 (24.1)	10 (13.5)	26 (11.8)	5 (4.3)	58 (16.4)	15 (7.9)
Any common mental disorder^a^	69 (52.7)	24 (32.4)	111 (51.4)	20 (17.1)	180 (51.9)	44 (23.0)
12-month prevalence						
Mood disorder	31 (23.3)	11 (14.9)	63 (28.6)	12 (10.3)	94 (26.6)	23 (12.0)
Anxiety disorder^a^	37 (29.8)	7 (9.5)	80 (43.5)	17 (14.5)	117 (38.0)	24 (12.6)
Substance use disorder	44 (33.1)	14 (18.9)	37 (16.8)	7 (6.0)	81 (22.9)	21 (11.0)
Any common mental disorder^b^	81 (61.8)	30 (40.5)	124 (57.7)	24 (20.5)	205 (59.2)	54 (28.3)
Lifetime prevalence						
Mood disorder	51 (38.3)	15 (20.3)	110 (50.0)	21 (17.9)	161 (45.6)	36 (18.8)
Anxiety disorder^a^	41 (35.3)	10 (13.5)	95 (53.1)	29 (24.8)	136 (46.1)	39 (20.4)
Substance use disorder	90 (67.7)	23 (31.1)	92 (41.8)	21 (17.9)	182 (51.6)	44 (23.0)
Any common mental disorder^b^	110 (84.6)	38 (51.4)	162 (75.7)	45 (38.5)	272 (79.1)	83 (43.5)

a. Those with post-traumatic stress disorder (PTSD) as their only anxiety diagnosis have been excluded as they have trauma by definition; the denominators for those with trauma were as follows: men *n* = 124, women *n* = 187, total *N* = 311 for current anxiety prevalence; men *n* = 124, women *n* = 184, total *N* = 308 for 12-month anxiety prevalence; men *n* = 116, women *n* = 179, total *N* = 295 for lifetime anxiety prevalence. The denominators for those without trauma were unaffected by removal of patients with PTSD only.

b. Those with PTSD as their only diagnosis have been excluded as they have trauma by definition; the denominators for those with trauma were as follows: men *n* = 131, women *n* = 216, total *N* = 347 for current common mental disorder (CMD) prevalence; men *n* = 131, women *n* = 215, total *N* = 346 for past 12-month CMD prevalence; men *n* = 130, women *n* = 214, total *N* = 344 for lifetime CMD prevalence. The denominators for those without trauma were unaffected by removal of patients with PTSD only.

### Associations between risk factors and CMDs

The results of univariate logistic regression analyses, examining associations between exposures and a diagnosis of an anxiety disorder, mood disorder, substance use disorder and any CMD in the past 12 months, are presented in Table 2. Variable categories and descriptions have previously been described.^12^ Additionally, for the purposes of this study, a specific variable with sexual and physical violence event categories was created, using four levels: no sexual or physical violence (reference level), sexual violence only, physical violence only and both physical and sexual violence experienced. Variables with a *P*-value ≤ 0.1 were carried forward into the multivariate logistic regression analysis (adjusted analyses). Results before and after the stepwise regression are both presented.

**Table 2:** Univariate logistic regression analyses for the association of trauma exposure with a diagnosis of a common mental disorder in the past 12 months

Parameters	Anxiety disorder			Mood disorder			Substance use disorder			Any common mental disorder		
	Odds ratio	95% CI	*P-*value	Odds ratio	95% CI	*P*-value	Odds ratio	95% CI	*P-*value	Odds ratio	95% CI	*P-*value
Age at interview	1.00	0.99–1.01	0.986	1.00	0.99–1.02	0.701	0.98	0.97–1.00	0.025	0.99	0.98–1.00	0.142
Gender												
Female	1.66	1.11–2.53	0.016	1.13	0.74–1.73	0.588	0.39	0.25–0.60	<0.001	0.68	0.48–0.97	0.031
Male	−	−	−	−	−	−	−	−	−	−	−	−
Childhood trauma												
Age <10 years	3.06	1.96–4.78	<0.001	2.62	1.69–4.06	<0.001	1.81	1.13–2.87	0.013	2.51	1.67–3.80	<0.001
Age ≥10 years	−	−	−	−	−	−	−	−	−	−	−	−
Region												
Metro	0.86	0.48–1.46	0.577	1.58	0.86–3.07	0.162	0.74	0.43–1.33	0.295	1.00	0.63–1.62	0.987
Rural	−	−	−	−	−	−	−	−	−	−	−	−
Non-interpersonal trauma												
Yes	1.54	1.01–2.34	0.042	1.30	0.84–2.00	0.231	1.38	0.87–2.16	0.162	1.55	1.07–2.25	0.020
No	−	−	−	−	−	−	−	−	−	−	−	−
Unexpected death of a loved one												
Yes	2.05	1.35–3.12	0.001	2.08	1.35–3.18	0.001	1.42	0.89–2.22	0.136	2.48	1.70–3.65	<0.001
No	−	−	−	−	−	−	−	−	−	−	−	−
Sexual and/or physical violence experienced												
Both	5.32	2.84–10.07	<0.001	5.99	3.35–10.80	<0.001	1.68	0.85–3.19	0.064	4.49	2.52–8.29	<0.001
Physical violence only	2.32	1.42–3.78		2.08	1.23–3.48		1.94	1.17–3.22		2.14	1.41–3.27	
Sexual violence only	5.24	2.66–10.47		2.25	1.04–4.60		1.39	0.60–2.97		3.81	1.98–7.67	
None	−	−	−	−	−	−	−	−	−	−	−	−
Comorbid PTSD												
Yes				6.83	4.15–11.34	<0.001	1.85	1.07–3.14	0.025	5.40	3.06–10.12	<0.001
No				−	−	−	−	−	−	−	−	−
Any trauma												
Yes	4.26	2.66–7.06	<0.001	2.65	1.64–4.44	0.001	2.41	1.46–4.13	<0.001	3.69	2.53–5.43	<0.001
No	−	−	−	−	−	−	−	−	−	−	−	−

Participants with post-traumatic stress disorder (PTSD) only, *n* = 45.

Separate multiple regression models were used with ‘any’ trauma (Table 3) and trauma subtypes (Table 4) as predictors, to examine associations between a diagnosis of anxiety disorder, mood disorder, substance use disorder and any CMD (in the presence of other predictors) in the past 12 months. Those with any trauma experienced a 4.01-fold (95% CI 2.72–5.97) increased risk of a diagnosis of any CMD (Table 3). Those with any trauma also experienced a 4.38-fold (95% CI 2.73–7.28), 2.65-fold (95% CI 1.64–4.44) and 2.78-fold (95% CI 1.65–4.85) increased risk of a diagnosis of an anxiety disorder, mood disorder or substance use disorder, respectively (Table 3). When investigating trauma exposures, comorbid PTSD was associated with a 4.53-fold (95% CI 2.61–7.92) increased risk of a mood disorder, a 2.47-fold (95% CI 1.38–4.35) increased risk of substance use disorder and a 3.58-fold (95% CI 1.90–7.06) increased risk of any CMD (Table 4). Women were much less likely to experience a substance use disorder (odds ratio 0.34, 95% CI 0.21–0.53) than men, and had an almost halved lower risk of any CMD (odds ratio 0.49, 95% CI 0.33–0.72) (Table 4). History of physical violence, and particularly sexual violence (odds ratio 3.05, 95% CI 1.51–6.41) or both physical and sexual violence (odds ratio 3.14, 95% CI 1.64–6.16), were associated with increased risks of a diagnosis of anxiety disorder, mood disorder or any CMD in the past 12 months (*P* < 0.001, *P* = 0.015 and *P* < 0.001, respectively; Table 4). The unexpected death of a loved one also had a significant effect on the odds of having an anxiety disorder (odds ratio 1.65, 95% CI 1.05–2.56), mood disorder (odds ratio 1.68, 95% CI 1.05–2.67) or any CMD (odds ratio 2.06, 95% CI 1.37–3.13) (Table 4).

**Table 3:** Multivariate logistic regression analysis for association of exposures/predictors, including overall trauma, with a diagnosis of a common mental disorder in the past 12 months

Parameters	Anxiety disorder			Mood disorder			Substance use disorder			Any common mental disorder		
	Odds ratio	95% CI	*P*-value	Odds ratio	95% CI	*P*-value	Odds ratio	95% CI	*P*-value	Odds ratio	95% CI	*P*-value
Before stepwise regression												
Age at interview	1.00	0.98–1.01	0.498	1.00	0.99–1.01	0.989	0.98	0.96–0.99	0.007	0.99	0.97–1.00	0.019
Gender												
Female	1.77	1.16–2.73	0.009	1.11	0.72–1.72	0.634	0.37	0.24–0.58	<0.001	0.65	0.44–0.93	0.020
Male	−	−	−	−	−	−	−	−	−	−	−	−
Region												
Metro	0.97	0.53–1.70	0.904	1.47	0.80–2.90	0.238	0.69	0.39–1.28	0.224	0.89	0.54–1.48	0.666
Rural	−	−	−	−	−	−	−	−	−	−	−	−
Any trauma												
Yes	4.44	2.76–7.41	<0.001	2.61	1.61–4.39	<0.001	2.84	1.68–4.97	<0.001	4.03	2.74–6.01	<0.001
No	−	−	−	−	−	−	−	−	−	−	−	−
After stepwise regression												
Age at interview							0.98	0.96–0.99	0.007	0.99	0.97–1.00	0.020
Gender												
Female	1.77	1.16–2.74	0.009				0.37	0.23–0.58	<0.001	0.64	0.44–0.93	0.020
Male	−	−	−				−	−	−	−	−	−
Any trauma												
Yes	4.38	2.73–7.28	<0.001	2.65	1.64–4.44	<0.001	2.78	1.65–4.85	<0.001	4.01	2.72–5.97	<0.001
No	−	−	−	−	−	−	−	−	−	−	−	−

**Table 4:** Multivariate logistic regression analysis for association of exposures/predictors, including trauma subtypes, with a diagnosis of a common mental disorder in the past 12 months

Parameters	Anxiety disorder			Mood disorder			Substance use disorder			Any common mental disorder		
	Odds ratio	95% CI	*P*-value	Odds ratio	95% CI	*P*-value	Odds ratio	95% CI	*P*-value	Odds ratio	95% CI	*P*-value
Before stepwise regression												
Age at interview							0.98	0.96–1.00	0.013			
Gender												
Female	1.50	0.97–2.35	0.071				0.34	0.21–0.55	<0.001	0.49	0.33–0.72	<0.001
Male	−	−	−				−	−	−	−	−	−
Childhood trauma												
Age <10 years	1.48	0.86–2.56	0.155	1.09	0.61–1.93	0.762	1.29	0.71–2.33	0.393	1.01	0.59–1.70	0.976
Age ≥10 years	−	−	−	−	−	−	−	−	−	−	−	−
Sexual and/or physical violence												
Both	3.59	1.75–7.39	<0.001	2.87	1.37–5.95	0.041	1.43	0.61–3.23	0.332	3.07	1.50–6.41	0.004
Physical violence only	1.91	1.11–3.27		1.34	0.74–2.37		1.72	0.96–3.06		1.48	0.91–2.39	
Sexual violence only	3.54	1.65–7.61		1.24	0.51–2.84		1.21	0.47–2.90		3.02	1.41–6.70	
None	−	−	−	−	−	−	−	−	−	−	−	−
Unexpected death of a loved one												
Yes	1.62	1.04–2.56	0.033	1.67	1.04–2.66	0.032				2.05	1.36–3.12	0.001
No	−	−	−	−	−	−				−	−	−
Non-interpersonal trauma												
Yes	1.12	0.70–1.78	0.629							1.07	0.70,1.63	0.745
No	−	−	−							−	−	−
Comorbid PTSD												
Yes				4.47	2.56–7.87	<0.001	1.87	0.98–3.52	0.053	3.56	1.88,7.07	<0.001
No				−	−	−	−	−	−	−	−	−
After stepwise regression												
Age at Interview							0.98	0.97–1.00	0.017			
Gender												
Female							0.34	0.21–0.53	<0.001	0.49	0.33–0.72	<0.001
Male							−	−	−	−	−	−
Sexual and/or physical violence												
Both	4.98	2.64–9.48	<0.001	3.02	1.55–5.81	0.015				3.14	1.64–6.16	<0.001
Physical violence only	2.11	1.28–3.46		1.37	0.77–2.38					1.51	0.96–2.37	
Sexual violence only	4.72	2.37–9.50		1.30	0.56–2.82					3.05	1.51–6.41	
None	−	−	−	−	−	−	−	−	−	−	−	−
Unexpected death of a loved one												
Yes	1.65	1.05–2.56	0.027	1.68	1.05–2.67	0.030				2.06	1.37–3.13	0.001
No	−	−	−	−	−	−				−	−	−
Comorbid PTSD												
Yes				4.53	2.61–7.92	<0.001	2.47	1.38–4.35	0.002	3.58	1.90,7.06	<0.001
No				−	−	−	−	−	−	−	−	−

PTSD, post-traumatic stress disorder.

## Discussion

It is largely acknowledged that Indigenous Australians experience more trauma than the general Australian population, because of past and present disadvantages and discrimination. A significant proportion of the trauma experienced by Indigenous Australians is culturally specific, such as forced removal from traditional homeland (‘country’), mass removal of children from parents (‘taken’), systemic racism, discrimination and greater disadvantage than any other population group. Much of this trauma is not captured by Western assessment tools. Currently available data are largely limited to specific populations or are self-reported. For instance, an Australian study conducted with the World Health Organization Composite International Diagnostic Interview identified an average of 2.9 events per person in 331 Indigenous men in custody,^15^ which doubled for those with a diagnosis of PTSD in the same survey. Similarly, a community-based study reported lifetime exposure to a traumatic event among 97.3% of Aboriginal participants from three communities in Western Australia (*N* = 221).^16^ In a representative community sample that used a clinical interview, Indigenous adults reported a 62.6% prevalence of traumatic life events, which is not increased, compared with other populations. The prevalence of four common trauma categories, however, were 1.7- to 3.0-times higher than in the Australian population,^12^ with the rate doubling in those with PTSD.^12^

To our knowledge, this is the first study to report on the prevalence of trauma for those with a CMD by using a clinical structured interview in a representative cohort of Indigenous Australians. In this Indigenous Australian cohort, we found that the presence of trauma for those with a lifetime prevalence of CMDs was 64.9%. Among these, 79.1% had a lifetime prevalence of a CMD, compared with 43.5% reporting a CMD but no trauma. The prevalence of any trauma is associated with almost twice the risk, for those with a CMD. Our study has also shown that the presence of harmful traumas increases the risk of having another CMD by 4.01-fold. Sexual assault was shown to increase prevalence of CMDs four-fold. This ratio has been similar to seminal work investigating the effects of sexual assault within marginalised populations.^17^ The high rates of violence and sexual assault within Australian Indigenous populations have been well-documented, and having strong culture and community is purported as a key strategy to mitigate violence.^18^

Our study found that trauma exposure was associated with a higher prevalence of CMDs in a representative Indigenous Australian population. Trauma exposure was associated with a 4.38-, 2.65- and 2.78-fold increased risk of having an anxiety disorder, mood disorder or substance use disorder, respectively. The presence of comorbid PTSD and trauma exposure was further associated with a 4.53-fold increased risk of a diagnosis of mood disorder in the past 12 months and a 3.58-fold increased risk of a diagnosis of any CMD in the past 12 months. This study also shows that Indigenous Australians who have experienced trauma exposure are 4.01 times more likely to have a diagnosis of any CMD in the past 12 months. In our previous report on this cohort, we identified that there was a stark increased rate of a current CMD compared with a 12-month or lifetime prevalence of a diagnosis.^11^ This shows that past events may have a lower recall rate, and that only severe or specific types of traumas are more likely to be remembered.

Despite a general agreement of the high prevalence of mental health disorders among Indigenous populations, nationally representative, mental health surveys that use face-to-face diagnostic interviews have not measured CMD rates in Indigenous populations, although smaller studies have used self-reported symptoms or specific population samples.^19,20^ Notably, the 2007 National Survey of Mental Health and Wellbeing, a study considered to be a benchmark prevalence study for mental health in Australia, did not collect data on the Indigenous status of participants.^21^ Another national prevalence study specifically assessing the mental health of children, the Young Minds Matter study, specifically excluded Indigenous children.^22^ As a result, the National Aboriginal Torres Strait Islander Social Survey (NATSISS), conducted in 2014–2015 by the Australian Bureau of Statistics, is the only published nationally representative study.^5^ However, this study did not use a diagnostic assessment, but asked participants if a doctor or nurse had informed them of having depression, anxiety, substance use disorder, or behavioural or emotional mental health conditions. The NATSISS identified that 29% of Indigenous people aged >15 years reported that they had a mental health condition.

Our previous study used a culturally acceptable diagnostic tool (the SCID-I) to assess the prevalence of CMDs^23^ in 544 Indigenous adults over a wide geographical area. We found that the standardised prevalence rates of mood, anxiety and substance use disorders were 6.7-, 3.8- and 6.9-fold higher^11^ than the general population. The crude rates of a current, 12-month and lifetime prevalence of PTSD were 13.8%, 15.3% and 20.8%, respectively – the highest prevalence rates for any individual CMD in our study.

In further work investigating the prevalence of traumatic life events and the risk of developing PTSD, we found that 64.9% of participants reported a lifetime trauma.^12^ The standardised prevalence of a PTSD diagnosis in the past 12 months was 13.3% (95% CI 10.4–16.1) overall, 16.1% (95% CI 12.2–19.9) in women and 8.2% (95% CI 5.3–11.1) in men; three times higher than the Australian reported rates.^12^ However, this study did not investigate the association of trauma with other CMDs.

Experiencing trauma has been significantly widespread and recurring across generations for Indigenous Australians;^6^ the risk of developing a CMD for those having experienced traumatic exposures is considerably exacerbated. As a consequence of social injustices and ongoing systemic disadvantages, the interaction of past and present traumas often present themselves as a collective trauma.^7^

### Limitations

Although this study incorporates a large broadly representative sample of Indigenous Australians by Indigenous mental health research standards, the study may not be adequately powered to determine accurate associations of CMDs and trauma, or specifically representative of the whole Australian Indigenous population. Likewise, the data does not account for exposures not readily identified with Western assessment tools that are unique to this population; for example, the impacts of disrupted cultural identity development and experiences of direct and institutional racism, which may provide more nuanced views of trauma exposure. Larger more diverse studies of this kind are further required to determine a more accurate and descriptive analysis of the prevalence of trauma and risk of CMDs within this population.

Another limitation is that the diagnoses made in this study were based on assessments that used the DSM-IV-TR criteria for trauma and CMDs, which have been updated in the newer DSM-5 version.^24^ The DSM-5 includes revisions to the symptom clusters and a more explicit definition of trauma, with specific examples; it is likely that applying the DSM-5 criteria of trauma to the data used in this study may have produced different outcomes. Furthermore, despite the DSM-IV being shown to be culturally acceptable,^23^ the DSM is a Western classification system and the same constructs and diagnostic criteria may not be applicable for Indigenous Australians.^25^ The presence of recall bias may also be present, as the assessments were conducted retrospectively. Similarly, there may also be an influence of the Indigenous concept of ‘shame’^26^ on the diagnoses being made; this concept largely stems from historical breaches of confidentiality and trust, and the compounding negative effects of racial disadvantage resulting from past segregation policies, displacement and separation of families.

Findings from this study suggest that the presence of debilitating trauma in this Indigenous cohort greatly increased the risk of developing other CMDs. There needs to be consideration of the underlying social determinants that lead to trauma exposure and risk of CMDs. Without addressing these concerns as a priority, the urgent need to break intergenerational cycles of trauma affecting social and emotional well-being of Indigenous Australians cannot be addressed. It is essential to address these social issues and focus on mitigating trauma, to reduce the burden of disease represented by poor mental health. Closing existing gaps contributing to the mental health of Indigenous Australians is an urgent priority, and there is strong evidence for it to be a specifically targeted national priority.

## Data availability

The University of Queensland Human Ethics Committee imposes restrictions on the data. Anonymised data are available to researchers who meet the conditions of the ethics approval and research governance policy that applies to this study via UQ eSpace. Requests for the data may be sent to the lead author, B.F.N.

## Appendix

**Appendix 1:** Standardised prevalence of common mental disorders in Indigenous Australians reporting trauma exposure (95% confidence intervals)

	Trauma (*n* = 353)	No trauma (*n* = 191)
30-day prevalence		
Mood disorder	21.96 (17.15–27.66)	11.85 (6.93–18.86)
Anxiety disorder^a^	36.15 (28.80–44.65)	13.19 (8.18–20.10)
Substance use disorder	16.16 (11.97–21.26)	9.66 (5.28–16.12)
Any common mental disorder^a^	52.59 (44.23–61.93)	25.54 (18.39–34.49)
12-month prevalence		
Mood disorder	26.57 (21.01–33.07)	14.88 (9.32–22.49)
Anxiety disorder^a^	40.73 (32.83–49.79)	14.08 (8.91–21.09)
Substance use disorder	22.40 (17.52–28.16)	12.96 (7.88–20.01)
Any common mental disorder^a^	60.38 (51.38–70.36)	30.33 (22.59–39.81)
Lifetime prevalence		
Mood disorder	43.76 (36.72–51.68)	22.01 (15.18–30.80)
Anxiety disorder^a^	47.92 (39.40–57.59)	21.13 (14.89–29.06)
Substance use disorder	49.83 (42.18–58.37)	23.09 (16.62–31.19)
Any common mental disorder^a^	78.97 (68.90–90.00)	43.41 (34.35–54.09)

Standardised prevalence rates were calculated using direct-age standardisation. The 2001 Australian General Population standard was used as the standard population to compare against. Because of small counts, we sometimes had to combine age groups to obtain estimates that are more reliable. Despite these groupings, some of the denominators were still below the recommended 30,^14^ and so caution should be taken when interpreting these estimates. The 95% confidence intervals were calculated with the Dobson method.^27^

a. Those with only a diagnosis of post-traumatic stress disorder were excluded.
